# Expanding the clinical and molecular spectrum of *ATP6V1A* related metabolic cutis laxa

**DOI:** 10.1002/jimd.12341

**Published:** 2021-02-04

**Authors:** Guido Vogt, Naji El Choubassi, Ágnes Herczegfalvi, Heike Kölbel, Anja Lekaj, Ulrike Schara, Manuel Holtgrewe, Sabine Krause, Rita Horvath, Markus Schuelke, Christoph Hübner, Stefan Mundlos, Andreas Roos, Hanns Lochmüller, Veronika Karcagi, Uwe Kornak, Björn Fischer‐Zirnsak

**Affiliations:** ^1^ Institut für Medizinische Genetik und Humangenetik, Charité ‐ Universitätsmedizin Berlin, corporate member of Freie Universität Berlin, Humboldt Universität zu Berlin, and Berlin Institute of Health Berlin Germany; ^2^ Max Planck Institute for Molecular Genetics, RG Development & Disease Berlin Germany; ^3^ Department of Pediatric Neurology Semmelweis Medical University, II. Pediatric Clinic Budapest Hungary; ^4^ Department of Pediatric Neurology University Hospital Essen, University Duisburg‐Essen Essen Germany; ^5^ CUBI – Core Unit Bioinformatics Berlin Institute of Health Berlin Germany; ^6^ Friedrich‐Baur‐Institute, Department of Neurology Ludwig‐Maximilians‐University of Munich Munich Germany; ^7^ Department of Clinical Neurosciences University of Cambridge, Cambridge Biomedical Campus Cambridge UK; ^8^ Department of Neuropediatrics, Charité‐Universitätsmedizin Berlin corporate member of Freie Universität Berlin, Humboldt‐Universität zu Berlin, and Berlin Institute of Health Berlin Germany; ^9^ Children's Hospital of Eastern Ontario Research Institute University of Ottawa Ottawa Ontario Canada; ^10^ Division of Neurology, Department of Medicine, The Ottawa Hospital Ottawa Canada; ^11^ Brain and Mind Research Institute University of Ottawa Ottawa Canada; ^12^ NIEH, Department of Molecular Genetics and Diagnostics Budapest Hungary; ^13^ Istenhegyi Genetic Diagnostic Centre Budapest Hungary; ^14^ Institute of Human Genetics University Medical Center Göttingen Göttingen Germany

**Keywords:** *ATP6V1A*, autosomal recessive cutis laxa, Golgi apparatus, hypotonia, progeroid features, v‐ATPase

## Abstract

Several inborn errors of metabolism show cutis laxa as a highly recognizable feature. One group of these metabolic cutis laxa conditions is autosomal recessive cutis laxa type 2 caused by defects in v‐ATPase components or the mitochondrial proline cycle. Besides cutis laxa, muscular hypotonia and cardiac abnormalities are hallmarks of autosomal recessive cutis laxa type 2D (ARCL2D) due to pathogenic variants in *ATP6V1A* encoding subunit A of the v‐ATPase. Here, we report on three affected individuals from two families with ARCL2D in whom we performed whole exome and Sanger sequencing. We performed functional studies in fibroblasts from one individual, summarized all known probands' clinical, molecular, and biochemical features and compared them, also to other metabolic forms of cutis laxa. We identified novel missense and the first nonsense variant strongly affecting *ATP6V1A* expression. All six ARCL2D affected individuals show equally severe cutis laxa and dysmorphism at birth. While for one no information was available, two died in infancy and three are now adolescents with mild or absent intellectual disability. Muscular weakness, ptosis, contractures, and elevated muscle enzymes indicated a persistent myopathy. In cellular studies, a fragmented Golgi compartment, a delayed Brefeldin A‐induced retrograde transport and glycosylation abnormalities were present in fibroblasts from two individuals. This is the second and confirmatory report on pathogenic variants in *ATP6V1A* as the cause of this extremely rare condition and the first to describe a nonsense allele. Our data highlight the tremendous clinical variability of *ATP6V1A* related phenotypes even within the same family.

**Synopsis**

This study expands our knowledge about *ATP6V1A* related cutis laxa and the postnatal cardiac decompensation leading to death of some individuals stresses the fact that these patients need neonatal intensive care.

## 1 INTRODUCTION

Cutis laxa (CL) is a phenotypic feature present in different rare genetic multisystem diseases. Affected individuals show lax, wrinkled and sometimes thin skin due to a reduction of dermal elastic fibers leading to a progeroid appearance. In addition, some CL conditions show metabolic changes such as alterations affecting cellular copper levels, protein glycosylation, and mitochondrial amino acid metabolism.^1^, ^2^, ^3^, ^4^, ^5^, ^6^, ^7^ Two examples are the conditions due to pathogenic variants affecting the mitochondrial components PYCR1 (ARCL2B; OMIM: 612940 and ARCL3B 614 438) and P5CS (ARCL3A; OMIM 219150 and ADCL3; OMIM: 616603). The affected individuals show an overall progeroid appearance, a typical triangular facial gestalt with cataracts, contractures, and in some lowered levels of plasma proline, arginine, or ornithine.^3^, ^4^, ^6^ Another group are the conditions showing altered serum protein glycosylation due to pathogenic variants affecting components of the highly conserved vacuolar‐type H^+^‐ATPases. These are large protein complexes, composed of two multicomponent sectors, V_0_ and V_1_ fulfilling diverse physiological functions. The V_0_ sector is membrane embedded and is responsible for proton translocation across the membrane. V_0_ is composed of a proteolipid ring of 10 c and individual a, d, e, ap1 and ap2 subunits. The peripheral V_1_ sector is composed of three A, B2, E1, G2, and single C1, D, and F subunits. Its function is ATP hydrolysis providing energy for the rotation of the central rotor subcomplex thereby facilitating proton translocation through the membrane via the V_0_ sector.^8^ v‐ATPases are mainly involved in the regulation of cellular pH homeostasis and in acidification of intracellular compartments such as endosomes, lysosomes, the Golgi apparatus, and secretory vesicles. Additionally, they play several roles in endocytosis and intracellular trafficking processes and regulate intracellular signaling pathways.^9^

Pathogenic variants affecting different v‐ATPase components lead to various forms of rare Mendelian disorders ranging from severe osteopetrosis over renal acidosis conditions to the diseases associated with cutis laxa.^5^, ^10^, ^11^ It has been shown that pathogenic variants in *ATP6V0A2*, encoding the a2 subunit of the v‐ATPase V_0_ sector, cause autosomal recessive cutis laxa type 2A (ARCL2A; OMIM: 219200) and its allelic disorder Wrinkly skin syndrome (WSS; OMIM: 278250).^5^ The affected individuals mainly show generalized cutis laxa, delayed closure of the anterior fontanel, and downslanting palpebral fissures. Neurologically, they show a variable degree of developmental delay, a cobblestone‐like brain malformation, seizures, and in some cases neurodegeneration. Biochemically, affected individuals present a congenital defect of glycosylation (CDG).^2^, ^5^, ^12^, ^13^ Additionally, pathogenic variants affecting the accessory v‐ATPase subunits ATP6AP1 (CDG2S; OMIM: 300972) and APT6AP2 (CDG2R; OMIM: 301045) have been shown as a cause of rare multisystem conditions with immunodeficiency and CL, associated with liver dysfunction and altered protein glycosylation.^14^, ^15^

In 2017, pathogenic variants in two other subunits of the v‐ATPase have been described to be causative for severely progeroid conditions with cutis laxa. Biallelic pathogenic variants in *ATP6V1E1*, encoding the E1 subunit of the V_1_ sector, have been shown to cause autosomal recessive cutis laxa type 2C (ARCL2C; OMIM: 617402). Besides congenital cutis laxa affected individuals show cardiac abnormalities, a triangular, mask‐like facial appearance and sometimes congenital cataracts, leading to a strong progeroid appearance.^1^, ^16^ Pathogenic variants affecting *ATP6V1A*, encoding the A subunit of the V_1_ sector have been shown as the cause of autosomal recessive cutis laxa type 2D (ARCL2D; OMIM: 617403). These affected individuals also show strong cutis laxa with redundant skin at birth. The facial appearance is similar to ARCL2C affected individuals.^1^, ^17^ Some affected individuals present with cardiac abnormalities and seizures.^1^ Additionally, in affected individuals with pathogenic variants in *ATP6V1E1* and *ATP6V1A* alterations of serum transferrin glycosylation as well as a reduced number of ICAM‐1 positive cells have been described previously classifying these conditions as a CDG.^1^

In the present study, we report on the clinical and molecular features of three affected individuals with a severely progeroid form of congenital cutis laxa with generalized muscular hypotonia.

## 2 AFFECTED INDIVIDUALS AND METHODS

### 2.1 Affected individuals

In this study, we report on two affected individuals from a Hungarian and an affected boy from a Turkish family with a severe cutis laxa phenotype at birth. From proband A‐II‐2, a skin biopsy was taken, and dermal fibroblasts were cultivated according to standard procedures. DNA from all affected individuals was isolated from peripheral blood samples.

### 2.2 Whole exome sequencing and co‐segregation

#### 2.2.1 Affected individual A‐II‐2

Enrichment for whole exome sequencing (WES) was carried out using the SureSelect XT, Clinical research exome V6 (Ilumina). Next generation sequencing was performed on a HiSeq 4000 (Ilumina). The reads were aligned using BWA‐MEMv0.7.15 to the reference GRCh37 (hs37d5.fa), separate read groups were assigned for all reads from one lane, and duplicates were masked using Samblaster v0.1.24.^18^ Standard QC was performed using FastQC. The variants were called using GATK Unified Genotyper v3.7 and were filtered using Varfish^19^ and MutationDistiller.^20^ Due to the clinical diagnosis, we used for variant interpretation a virtual gene panel for all genes associated with cutis laxa and removed variants with a population frequency above 1% found in gnomAD and the 1000 genomes project (TGP). Using this approach, we detected two suspicious variants in *ATP6V1A*. In silico predictions of pathogenicity were performed by Mutation Taster,^20^, ^21^ Polyphen‐2,^22^ and MetaDome.^23^ All variants were classified by the ACMG guidelines.^24^ Sanger sequencing was performed of *ATP6V0A2* (NM_012463.4) and pathogenic variants have been excluded in the affected individual A‐II‐2.

#### 2.2.2 Affected individual B‐II‐1

WES was performed by the Genomics Platform at the Broad Institute of MIT and Harvard, Cambridge, USA. Libraries were created with an Illumina exome capture (38 Mb target) and sequenced with a mean target coverage of >80x. Genomic and phenotypic data were submitted to the RD‐Connect Genome‐Phenome Analysis Platform, GPAP, (https://platform.rd-connect.eu), where they can be accessed under a controlled access agreement. Exome sequencing data were processed and analyzed on the RD‐Connect GPAP. Likely pathogenic variants were identified applying standard filtering for high to moderate variant effect predictor (ie, nonsense, splice site, frame‐shift, in‐frame and non‐synonymous variants), and for minor allele frequency < 1% in gnomAD (http://gnomad.broadinstitute.org), and in a cohort of 1182 ethnically matched Turkish control individuals (TUBITAK MAM‐GMBE dataset: http://gmbe.mam.tubitak.gov.tr/en). Shortlisted variants were interrogated for their predicted in silico deleteriousness, previous known association with human disease and were classified by the ACMG Guidelines.^24^

For co‐segregation analysis, the affected exons of *ATP6V1A* (NM_001690.3) were sequenced in the affected individuals and their parents when material was available. Sequencing reaction was performed using the BigDye Terminator cycle sequencing kit (ThermoFisher Scientific), and run on a 3730 DNA Analyzer (Applied Biosystems). Sequences were evaluated and compared to reference sequences using SeqMan (DNASTAR).

### 2.3 Muscle histology

Histological studies on the quadriceps biopsy derived from index patient B‐II‐1 were carried out as described before.^25^ Immunofluorescence studies on the same biopsy were performed following a recently published protocol^26^ utilizing a LAMP2 antibody (monoclonal mouse H4B4; kindly provided by Prof. M. Vorgerd).

### 2.4 Cell culture

Dermal fibroblasts from individual A‐II‐2 (obtained from the Muscle Tissue Culture Collection MTCC, Munich, Germany) and from control individuals were cultivated in DMEM (Lonza) supplemented with 10% fetal calf serum (Gibco), 1% UltraGlutamine (Lonza) and 1% penicillin/streptomycin (Lonza).

### 2.5 mRNA expression analysis

Cells were lysed in a confluent status with TRIzol (Invitrogen) and total RNA was prepared by a standard RNA extraction protocol. Total cDNA was reverse transcribed by RevertAid H Minus First Strand cDNA Synthesis Kit (Fermentas). Quantitative PCR was performed using EVAgreen (Solis BioDyne) on a QuantStudio 3 Real‐Time‐PCR System (ThermoFisher Scientific). Significance levels were calculated using Student's *t‐*test. Additionally, PCR amplicons of the regions affected by the pathogenic variants from cDNA were generated and subsequently sequences as described above. All primer sequences are available in Table S1.

### 2.6 Immunoblot

Cells were lysed with RIPA buffer (150 mM NaCl, 50 mM Tris, 5 mM EDTA, 1% Triton X‐100, 0.25% deoxycholate, and 0.1% SDS) supplemented with protease inhibitor (Complete, Roche). The 20 μg of protein per lane were separated by SDS‐PAGE and subsequently transferred to nitrocellulose membrane. After semidry blotting membranes were blocked 1 hour with blocking buffer (LI‐COR Biosciences) and probed with an antibody against ATP6V1A (1:1000, Abcam) and GAPDH (1:40 000, Ambion) overnight. Membranes were washed and incubated with HRP‐ or IRDye‐conjugated secondary antibodies. Signals were detected with Odyssey FC Imaging System and densitometric quantification was performed using Image Studio (LI‐COR Biosciences). Significance levels were calculated using Student's *t‐*test.

### 2.7 Brefeldin A‐induced Golgi collapse

Fibroblasts from the affected individual A‐II‐2 and appropriated controls were seeded on coverslips. The day after they were incubated with 5 μg/mL Brefeldin A (BFA) for 9 minutes and immediately fixed in 4% paraformaldehyde for 10 minutes at room temperature. Cells were washed three times in phosphate buffered saline (PBS) and afterward permeabilized and blocked with 0.1% saponin in 3% BSA for 20 minutes at room temperature. Immunofluorescence staining was performed for the Golgi markers GM130 (mouse anti‐GM130; BD Transduction Laboratories) and giantin (rabbit anti‐giantin; Covance) overnight in 3% BSA in 1x PBS. Cells were washed three times in PBS and secondary antibodies anti‐mouse IgG Alexa Fluor 555 (Invitrogen, Molecular Probes) and an anti‐rabbit IgG Alexa Fluor 488 (Invitrogen, Molecular Probes) were applied. DNA was stained by DAPI and cells were mounted in Fluoromount G (SouthernBiotech). Pictures were taken with a fluorescence microscope (BX60, Olympus). At least 100 cells per sample were counted and the experiment has been performed two times. Significance levels were calculated using Student's *t‐*test.

### 2.8 Metabolic labeling of ManNAz

Fibroblasts from the affected individual A‐II‐2 and appropriated controls were seeded in optical 96‐well plate. Additionally, control cells were preincubated with 2 μM tunicamycin (TN, Sigma Aldrich). Next day cells were incubated 6 hours with 50 μM tetraacetylated *N*‐azidoacetyl‐mannosamine (ManNAz, Jena Bioscience). Cells were washed three times with PBS, fixed 15 minutes with 4% PFA and permeabilised for 15 minutes with 0.5% Triton‐X‐100. After washing, cells were labeled with click reaction solution (10 μM Fluor 488‐Alkyne fluorescent probe/150 μM CuSO_4_x5H_2_O/300 μM BTTAA/2.5 mM ascorbic acid/100 mM K_2_HPO_4_, in water) for 1 hour and nuclei were stained by DAPI. The fluorescence intensities of the Golgi apparatus from individual cells were measured using Operetta High Content Imaging System (PerkinElmer). The experiment was performed two times. Significance levels were calculated using Student's *t‐*test.

## 3 RESULTS

### 3.1 Clinical presentation

**FIGURE 1:**
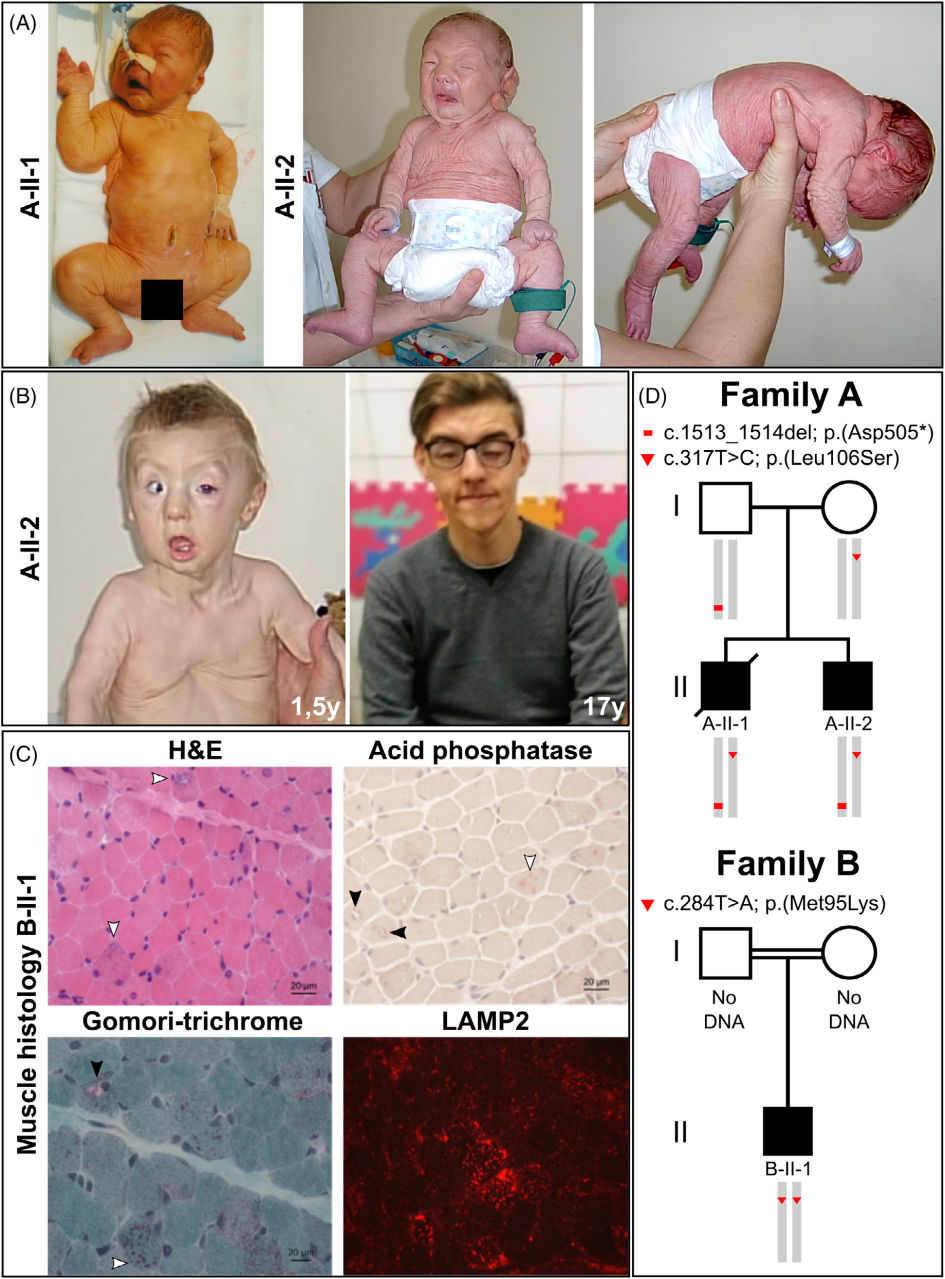
Clinical features of the affected individuals and disease gene identification. A, Both affected individuals A‐II‐1 and A‐II‐2 postnatally showed strong and generalized cutis laxa. Their facial gestalt was dysmorphic due to a triangular, mask‐like, face with a short and pointed chin. Additionally, individual A‐II‐2 showed a strong muscular hypotonia. B, Evolving phenotype in proband A‐II‐2 shows a strong improvement of the cutis laxa phenotype from 1.5 to 17 years of age. With 1.5 years of age he still showed an entropion and a strong *pectus excavatum* deformation. The cutis laxa phenotype improved within the first year of life. C, Histological findings: H&E staining revealed myofibres displaying centralized nuclei as well as rimmed vacuoles (white arrows). Additional Gomori‐trichrome staining shows mitochondrial accumulation within one representative vacuoles (black arrow) as well as within the sarcoplasm (white arrow) in a proportion of muscle cells. Acid phosphatase staining showed a positive reactivity of a proportion of vacuoles (black arrows) in addition to sarcoplasmic deposits (white arrow). Prominent build‐up of deposits of LAMP2 in a proportion of muscle fibers are respectively highlighted by white arrows. D, Schematic view of the co‐segregation analysis performed in both affected individuals from family A and their parents. The substitution c.317 T > C; p.(Leu106Ser) was transmitted via maternal, whereas the alteration c.1513_1514del; p.(Asp505*) was inherited through the paternal line. Both affected individuals were compound heterozygous. The alteration c.284 T > A; p.(Met95Lys) was found in a homozygous state in the affected individual from family B

The affected individuals A‐II‐1 and A‐II‐2 are the children of healthy, unrelated parents of Hungarian origin. Individual A‐II‐1 was born at 39 weeks of gestation with a weight of 3590 g. During pregnancy, decreased fetal movements were observed. At birth, he showed generalized cutis laxa with redundant skin and muscular hypotonia. Additionally, he showed a mask‐like facial expression and an overall progeroid appearance (Figure 1A). Postnatally, he suffered from respiratory distress and was ventilated for 8 days. He had a strong *pectus excavatum* and bilateral proximal and distal leg weakness and bilateral contractures of his hips and knees. A muscle biopsy revealed increased connective and fatty tissue, increased variability of fiber size, rounded fibers, some central nuclei and degenerating fibers with vacuoles (data not shown). Additionally, his creatine kinase (CK) was strongly elevated 13 000 to 15 000 U/L (ref. <190 U/L). His skin biopsy revealed a decrease of elastic fibers, collagen fibers with irregular shape and vacuolation of basal keratinocytes (data not shown). Chromosome analysis revealed a normal 46,XY karyotype. Additionally, molecular genetic diagnostic screening for pathogenic variants in *SMN1*, *GSDII*, *ATP6V0A2*, *PYCR1*, and *ALDH18A1* did not reveal any alterations. Additionally, transferrin isoelectric focusing did not show any protein glycosylation abnormalities. He died at 8 days of age due to cardiac and respiratory failure.

Individual A‐II‐2 was born after an uneventful pregnancy at 39 weeks of gestation with a weight of 3160 g (−0.75 SD), a length of 48 cm (−1.7 SD), and an OFC of 35.5 cm (+0.17 SD). At birth, he showed generalized cutis laxa with redundant skin, muscular hypotonia, and ptosis. A mask‐like, triangular facial appearance with a short forehead, hypertelorism and an entropion were evident. Due to respiratory distress, he was ventilated for 3 days after birth. He had joint laxity, sat with support and spoke a few words at 24 months and walked with support at 28 months of age. Heart ultrasound was normal at 18 months of age. Cranial MRI showed a thinning of the *corpus callosum*. A muscle biopsy revealed degenerating fibers with vacuoles implying a vacuolar myopathy in single fibers (data not shown) and his creatine kinase (CK) was constantly elevated 392 to 1082 U/L (ref. < 190 U/L). At 1.5 years of age, the cutis laxa did improve and he still showed a strong *pectus excavatum* deformity (Figure 1B). Overall, the phenotype improved with age and at 17 years of age, at the time of his last clinical evaluation, he still had a facial dysmorphism, showed thin skin and a weak muscle tone. However, his cognitive parameters were in a normal range and he attended high school. Chromosome analysis was performed and showed a normal 46,XY karyotype.

Individual B‐II‐1 is the first child of consanguineous parents of Turkish origin. After an uneventful pregnancy and normal birth, generalized muscular hypotonia, retrognathia and severe cutis laxa were recognized. The boy showed no respiratory distress after birth. Motor milestones were achieved in time (free sitting at 10 months of age, free walking at the age of 14 months). He showed normal speech and cognitive development. At the age of 4 years a neuropediatric consultation revealed general muscular hypotonia and weakness, a pectus carinatum, cutis laxa, and facial dysmorphism. Cranial MRI showed a cortical atrophy. Motor and sensory neurography showed flattened amplitudes of the muscle action potential and normal conduction velocities, thereby excluding a general neuropathy or significant α‐motoneuron decay. The serum creatine kinase level was elevated up to 1980 U/L (ref. <190 U/L). Up to now, several examinations excluded cardiac involvement. However, the boy was examined twice a year with pulmonary function test and developed a restrictive ventilatory problem with no acute need for ventilation. At the age of 14 years, he developed a severe scoliosis, a spine surgery was performed. His last visit at the age of 15 years revealed a normal endurance and muscle strength without any progression or loss of motor functions. His overall phenotype, especially his cutis laxa, improved with time and his cognitive parameters were still in a normal range. Genetic analysis showed no mutations in known genes for infantile myopathy.

### 3.2 Muscle histology from individual B‐II‐1

Histological examination of the quadriceps biopsy from individual B‐II‐1 revealed a pronounced pathology characterized by the presence of vacuoles of varying diameter (Figure 1C, Figure S1), whereas some of these vacuoles present empty, others are fuchsophil and/or show a reactivity for acid phosphatase (Figure 1C). Gomori‐trichrome showed a sarcoplasmic increase of mitochondria in a proportion of muscle fibers accompanied by accumulation within vacuoles (Figure 1C). In addition, NADH staining occasionally revealed focal sarcoplasmic decrease of enzymatic activity supporting the concept of irregular distribution of mitochondria in a proportion of muscle cells (Figure S1). The periodic acid‐Schiff (PAS) reaction revealed increase of deposits in a proportion of muscle fibers. Oil red staining showed increased lipid deposits in a proportion of muscle fibers (Figure S1). Additionally, immunofluorescence studies revealed increased actin immunoreactivity in a minority of myofibres. Myotilin, filamin C, and desmin showed focal increase of reactivity suggestive for accumulation of these structural proteins (Figue S1). Staining of LAMP2, a known marker for the build‐up of protein aggregates revealed a profound sarcoplasmic increase in the diseased muscle cells (Figure 1C). Of note, analysis on caveolin‐3 distribution showed—in addition to a regular sarcolemmal localization of the protein—a positive immunoreactivity of the rim of vacuoles (Figure S1).

### 3.3 Identification of the causative genetic defect

To elucidate the molecular genetic cause of the observed cutis laxa phenotype, we performed WES on the DNA from the affected individuals A‐II‐2 and B‐II‐1. In both samples we found suspicious variants in *ATP6V1A*. In the affected individual A‐II‐2, we found two heterozygous positions; however, the distance between them made it impossible to judge on a potential compound heterozygous situation from the WES data.

The variant c.317 T > C resides in exon 4 of *ATP6V1A* and is predicted to cause a substitution from leucin at position 106 of the ATP6V1A polypeptide to a serine p.(Leu106Ser). This substitution is absent from all control databases and predicted to be probably damaging by MutationTaster, Polyphen‐2, and Metadome (Table 1). The variant c.1513_1514del is a deletion of two nucleotides within exon 13, the penultimate exon of *ATP6V1A* predicted to cause a frameshift. The consequence is the immediate generation of a premature termination codon p.(Asp505*). This alteration is also absent from all control databases and predicted to be disease causing by MutationTaster (Table 1).

**TABLE 1:** Clinical features of affected individuals with biallelic *ATP6V1A* pathogenic variants

Affected individual	A‐II‐1	A‐II‐2	B‐II‐1	PIII:1^1^	PIV:1^1^	PV:1^1^					
Sex	Male	Male	Male	Male	Male	Male					
Ethnicity	Hungarian	Hungarian	Turkish	German	Pakistani	Turkish					
Consanguinous	No	No	Yes	No	Yes	Yes					
Age at last evaluation	8 days	17 years	15 years	15 years	3 months	?					
Reference	This study	This study	This study	van Damme et al.^1^				Phenotype comparison			
Clinical phenotype							Frequency	*ARCL2C*	*ARCL2A*	*ARCL2B*	*ARCL3A*
Generalized cutis laxa	+	+	+	+	+	+	6/6	+	+	+	+
Redundant skin	+	+	+	+	±	+	6/6	+	+	−	−
Improving cutis laxa with age	n.d.	+	+	+	n.d.	n.d.	3/6	n.d.	+	+	+
Facial dysmorphism^a^	+	+	+	±	+	+	6/6	+	+	+	+
Mask‐like facial appearance	+	+	+	+	+	+	6/6	+	−	−	−
Entropion	−	+	−	−	−	+	2/6	+	−	−	−
Hip dysplasia	−	−	−	−	+	−	1/6	+	+	−	−
Contractures	+	+	−	−	+	−	3/6	+	−	+	+
Pectus deformity	+	+	+	+	−	−	4/6	−	−	−	−
Muscular hypotonia	+	+	+	+	+	+	6/6	+	+	+	+
Cardiac abnormalities	Cardiac failure	−	−	+	+	+	4/6	+	−	−	−
Aortic dilatation	−	−	−	−	+	−	1/6	+	−	−	−
Seizures	−	−	−	+	+	n.d.	2/6	n.d.	+	−	−
Brain structural abnormalities	n.d.	+	+	+	+	±	5/6	−	+	+	+
Early demise	+	−	−	−	+	−	2/6	±	−	−	±
Cellular phenotype											
Serum glycosylation abnormalities	−	−	n.d.	±	±	n.d.	2/6	+	+	−	−
Altered Golgi morphology	n.d.	+	n.d.	+	n.d.	n.d.	2/6	+	+	−	−
Golgi trafficking defect	n.d.	+	n.d.	+	n.d.	n.d.	2/6	+	+	−	−
*ATP6V1A* (NM_001690.3) genotypes and in silico prediction								Affected gene			
cDNA	c.317 T > C/c.1513_1514del		c.284 T > A	c.1012C > T	c.215G > A	c.215G > A		*ATP6V1E1*	*ATP6V0A2*	*PYCR1*	*ALDH18A1*
Protein	p.(Leu106Ser)/p.(Asp505*)		p.(Met95Lys)	p.(Arg338Cys)	p.(Gly72Asp)	p.(Gly72Asp)					
Genotype	Compound heterozygous		Homozygous	Homozygous	Homozygous	Homozygous					
gnomAD Frequency	Absent/Absent		Absent	Absent	Absent	Absent					
Mutation Taster score	0.999 (DC)/0.999 (DC)		0.999 (DC)	0.999 (DC)	0.999 (DC)	0.999 (DC)					
Polyphen 2 score	1000 (PD)/−		0.754 (PD)	1000 (PD)	1000 (PD)	1000 (PD)					
Metadome	Intolerant/−		Neutral	Neutral	Slightly intolerant	Slightly intolerant					

*Note*: + present, − absent, ± might be present, n.d. not determined; ?, no information.

Abbreviations: DC, disease causing; PD, probably damaging.

^a^ Facial dysmorphism includes: triangular face, hypertelorism, pointed chin.

To investigate whether this variant segregates with the disease we performed co‐segregation analyses by Sanger sequencing using DNA samples from both affected individuals and their clinically unaffected parents. These analyses revealed the variant c.317 T > C; p.(Leu106Ser) to be inherited through the maternal, whereas the alteration c.1513_1514del; p.(Asp505*) was transmitted via the paternal line. In both affected individuals, we found both variants and can thus infer a compound heterozygous situation, in line with an autosomal recessive mode of inheritance (Figure 1D).

In individual B‐II‐1, we identified the variant c.284 T > A in a homozygous state. It also resides in exon 4 of *ATP6V1A* and is predicted to cause the substitution from methionine at position 95 of the ATP6V1A polypeptide to a lysin p.(Met95Lys) (Figure 1D). This substitution is absent from all control databases and predicted to be probably damaging by MutationTaster and Polyphen‐2. Unfortunately, no material from the clinically unaffected parents was available. All variants identified in the affected individuals have been classified as pathogenic (class V) according to the ACMG criteria.

**FIGURE 2:**
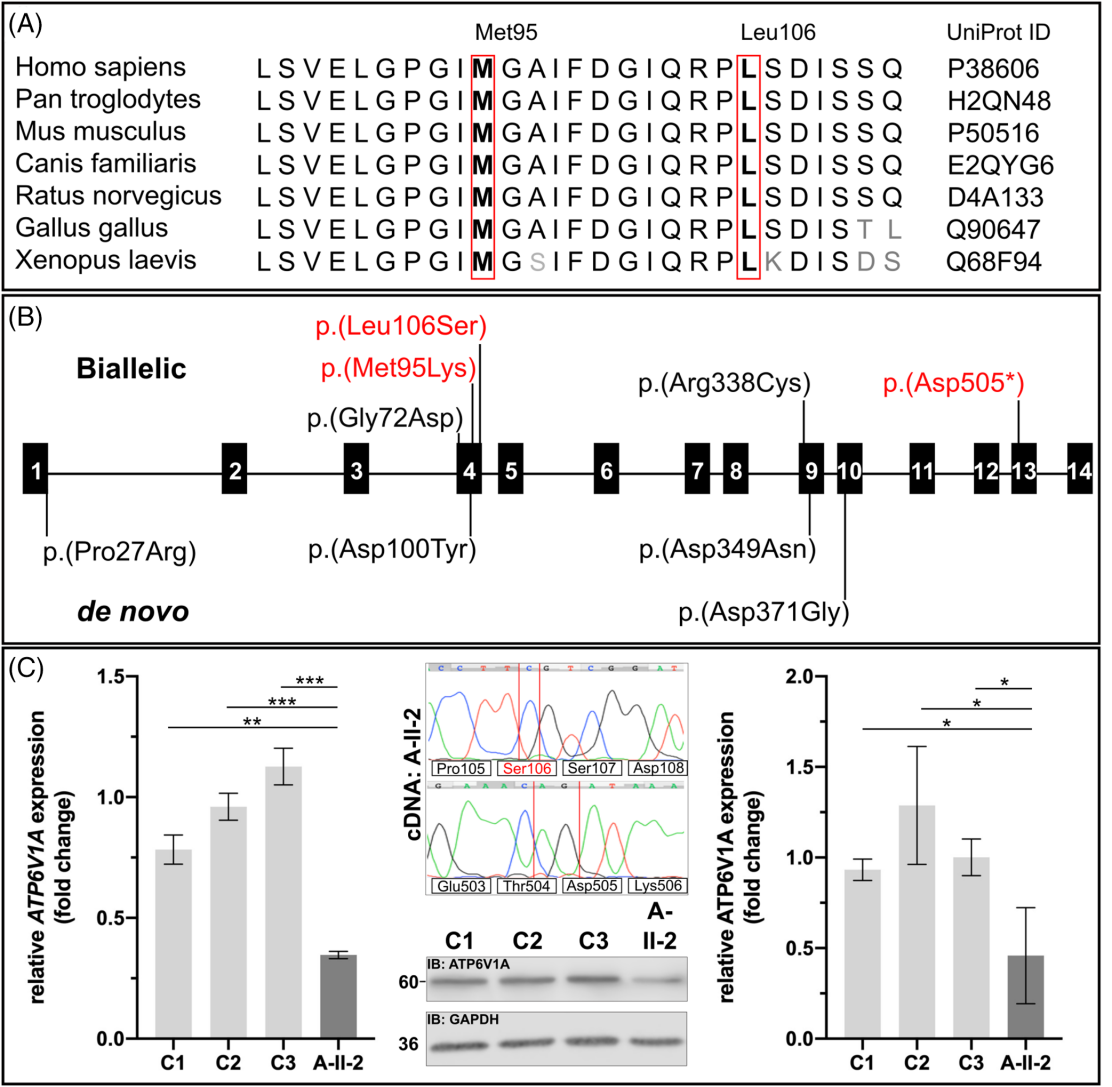
A, Conservation was investigated by an interspecies alignment. The residue Met95 and Leu106 are highly conserved down to *Xenopus laevis*. B, Schematic overview of all so far described alterations identified within *ATP6V1A*. Above the exonic structure all variants leading to ARCL2D are indicated including the two variants found in the present study (red). Below, all dominant de novo pathogenic variants, causative for epileptic encephalopathy are indicated. C, Functional consequence of the pathogenic variants in *ATP6V1A* in fibroblasts from individual A‐II‐2. Quantitative PCR was performed on cDNA extracted from fibroblasts from individual A‐II‐2 and controls. *ATP6V1A* expression was significantly reduced in the cells from proband A‐II‐2 (**P < .01; ***P < .0005). Amplification of *ATP6V1A* amplicons from individuals A‐II‐2 cDNA including the positions affected by the pathogenic variants. The two nucleotide deletion c.1513_1514del was absent on cDNA level while the variant c.317 T > C encoding the variant p.(Leu106Ser) was detectable. Analysis of ATP6V1A protein expression in individual A‐II‐2 fibroblasts revealed a significant reduction of protein expression by approximately 40% (*P < .05)

We investigated the conservation of the missense substitutions affecting Met95 and Leu106 and found them in a highly conserved (down to *Xenopus laevis*) part of the ATP6V1A polypeptide (Figure 2A). Two different pathogenic missense alleles, leading to ARCL2D have been described previously.^1^ Additionally, Fassio et al found de novo pathogenic missense variants causative for epileptic encephalopathy in the same gene.^27^ Mapping the different pathogenic alleles on the *ATP6V1A* genomic structure revealed no accumulation within a certain exon. However, the variant p.(Asp505*) is to our knowledge the first predicted nonsense allele identified in *ATP6V1A* (Figure 2B).

### 3.4 Functional studies in dermal skin fibroblasts

To further study the consequence of the identified variants, we performed quantitative RT‐PCR to investigate the expression of *ATP6V1A* and found in comparison to healthy controls the *ATP6V1A* mRNA reduced by approximately 65% in the cells from the affected individual A‐II‐2 (Figure 2C). To study this in more detail, PCR amplicons including the alterations were generated from cDNA of proband A‐II‐2. Subsequent Sanger sequencing revealed the allele carrying the missense alteration p.(Leu106Ser) to be expressed, whereas the allele harboring the nonsense alteration was not detectable (Figure 2C). Additionally, we investigated ATP6V1A protein levels using immunoblot analysis of the fibroblasts from proband A‐II‐2 in comparison to controls. Using an antibody against ATP6V1A, functionally validated in the literature,^1^ we found a band with the expected size in all individuals analyzed. However, the band intensity was strongly reduced in the lysates from the probands fibroblasts. Densitometric analyses from three independent experiments revealed a reduction of approximately 50% of band intensity proving a strong reduction of ATP6V1A in fibroblasts from the affected individual A‐II‐2 (Figure 2C).

**FIGURE 3:**
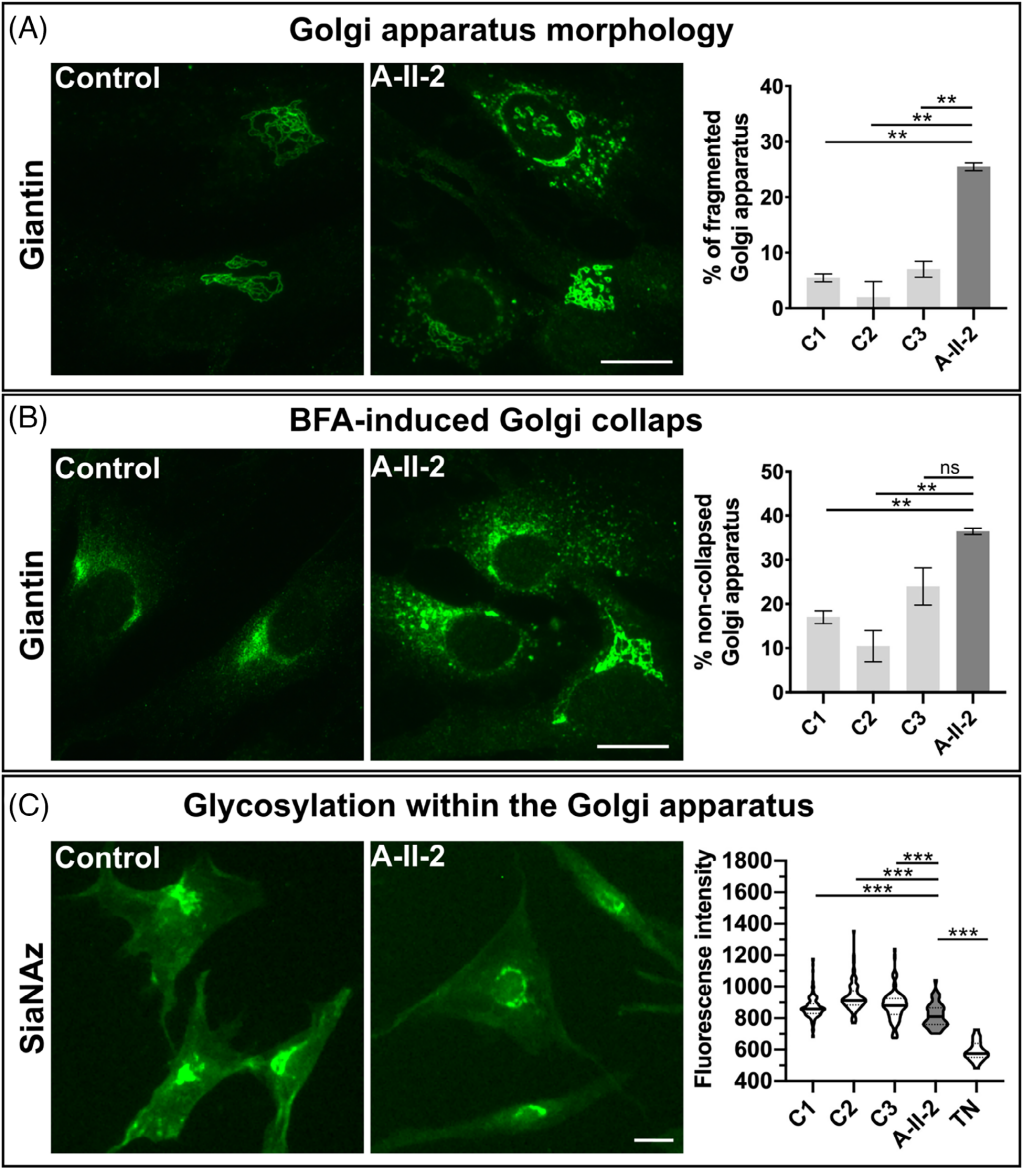
Abnormal function of the Golgi apparatus: A, Morphology of the Golgi apparatus was evaluated using immunofluorescence staining of Giantin. In control cells the Golgi apparatus showed regular cisternae whereas in proband A‐II‐2 fibroblasts the Golgi structure was dilated and fragmented. In controls below 10% of cells showed a fragmented and dilated Golgi, whereas in the cells from individual A‐II‐2 more than 25% of cells showed this fragmentation (**P < .01). Scale bar: 100 μm, B, Brefeldin A‐induced Golgi collapse. All cells were treated with BFA and Golgi collapse was measured in controls and cells from individual A‐II‐2 after 9 minutes. The Golgi collapse was significantly reduced in the proband's cells in comparison to controls (**P < .01=; ns = not significant. Scale bar: 100 μm, C, Glycosylation within the Golgi apparatus. Metabolic labelling of intra‐Golgi sialylation via the analysis of SiaNAz incorporation. In cells from individual A‐II‐2, we found a significant reduction of SiaNAz fluorescence indicating a glycosylation abnormality within the Golgi apparatus in comparison to controls (***P < .0005). Scale bar: 100 μm

Previously, it has been shown that loss‐of‐function pathogenic variants in *ATP6V0A2, ATP6V1A* and *ATP6V1E1* lead to changes in Golgi function.^1^, ^2^ In order to evaluate whether this was also true for our cases we stained the giantin protein to visualize the Golgi apparatus in fibroblasts from proband A‐II‐2 and matched controls. Golgi morphology was clearly altered with swollen and fragmented cisternae. Quantification revealed a fragmented Golgi apparatus in 26% of cells from the affected individual A‐II‐2 in comparison to less than 10% in controls (Figure 3A). Another phenomenon observed in fibroblasts from affected individuals carrying pathogenic variants in genes encoding v‐ATPase components (*ATP6V0A2*, *ATP6VE1*, and *ATP6V1A*) is a delayed retrograde transport from the Golgi apparatus to the endoplasmic reticulum (ER) transport induced by Brefeldin A (BFA) treatment.^1^, ^2^, ^5^ To study the impact of BFA on the fibroblasts from proband A‐II‐2, Golgi integrity was measured after 9 minutes BFA treatment. In control fibroblasts from three healthy individuals, below 25% of the cells had a noncollapsed Golgi apparatus after this treatment whereas in fibroblasts from individual A‐II‐2 36.5% showed a preserved Golgi structure (Figure 3B).

Given these alterations of the Golgi compartment we wanted to know whether protein glycosylation is altered in the fibroblasts from proband A‐II‐2. This was investigated using metabolic labelling of cells from individual A‐II‐2 and healthy controls with ManNAz to track sialylated glycol‐conjugate metabolism. ManNAz is taken up by the cell via an endocytosis independent mechanism and is intracellularly converted into SiaNAz (azido *N*‐acetylneuraminic acid). Subsequently, this structure is incorporated into proteins and lipids within the Golgi apparatus.^28^, ^29^ Using this method *N*‐azidoacetyl‐tagged sugars can be visualized by adding a fluorophore on each modified molecule. The global fluorescence intensity of the Golgi apparatus was measured using a high content screening system to assess the efficiency of SiaNAz incorporation. We found in healthy individuals fluorescence intensities in a range from 860 to 920 fluorescence units with an even distribution of single values. In fibroblasts from individual A‐II‐2, a significant reduction to 820 fluorescence units was detectable with a strong accumulation of values below the median of the controls. As a control experiment, we incubated control cells with tunicamycin (TN), a glycosylation inhibitor, and found a strong reduction of fluorescence intensity to 580 fluorescence units (Figure 3C).

## 4 DISCUSSION

In the present study, we identified pathogenic variants in *ATP6V1A* as the cause of a severe cutis laxa phenotype in affected individuals from two unrelated families. The observed phenotype with generalized cutis laxa, redundant skin and a typical mask‐like triangular face in all affected individuals is in line with the already published cases.^1^, ^17^ Interestingly, the referring clinicians first suspected ARCL2A due to facial similarity. It therefore seems that there is a suggestive facial appearance caused by mutations in *ATP6V0A2*, *ATP6V1E1*, and *ATP6V1A*. The two boys from family A showed postnatal respiratory distress and required ventilation for several days while the affected individual B‐II‐1 developed restrictive ventilatory problems later in life. The reason for this is currently unclear, but a delayed lung maturation or diaphragmatic weakness are possible explanations for the respiratory problems. Unfortunately, individual A‐II‐1 died at 8 days of age due to cardiac and respiratory failure. Individual PIV:1 described by van Damme et al also showed cardiac failure.^1^ Thus, respiratory and cardiac decompensation immediately after birth can lead to a drastic discordance of the clinical outcome, even in siblings. In contrast, the phenotype of individual A‐II‐2 and B‐II‐1 improved considerably after they had survived the postnatal period. A similar course was also described by van Damme and colleagues.^1^, ^17^ 2/6 known affected individuals died within the first days or months of life.^1^ However, after overcoming this critical period, the further development of the surviving individuals improved over time.

In comparison to *ATP6V0A2*‐related CL, where most affected individuals show a variable degree of intellectual disability, this feature is completely absent in the affected individual A‐II‐2 and B‐II‐1 from this study and has been not been previously described in others.^1^, ^17^ In *ATP6V0A2‐*related CL one consistent feature is a cobblestone malformation and many affected individuals show seizures. Also two‐third of the previously reported individuals with *ATP6V1A* pathogenic variants suffered from seizures and some also showed polymicrogyria and a thin *corpus callosum* (Table 1).^1^ In line with this, proband II‐2 also showed a thinning of the *corpus callosum* and individual B‐II‐1 showed cortical atrophy. However, they never developed seizures and are successful high school students.

The generalized muscular hypotonia described in affected individuals with pathogenic variants in *ATP6V1E1* and *ATP6V1A* has also been observed in the individuals A‐II‐1, A‐II‐2, and B‐II‐1 from this study (Table 1). The muscle biopsy revealed degenerating fibers with vacuoles characteristic for a metabolic myopathy. Additionally, histology revealed an increase and irregular distribution of muscle mitochondria implying a secondary mitochondrial dysfunction. Within the spectrum of CL conditions the differential diagnoses closest to ARCL2D are the forms due to pathogenic variants affecting the mitochondrial components PYCR1 and P5CS, all showing a progeroid appearance and strong muscular hypotonia (Table 1).^3^, ^4^, ^6^ However, also in *ATP6V0A2* and *ATP6V1E1* related cutis laxa, this is a common feature (Table 1).^30^ So far it is not clear how the Golgi apparatus and the mitochondria are interconnected. In all v‐ATPase related CL conditions the Golgi apparatus in patient derived cells shows an altered morphology. This includes a dilatation and/or fragmentation of this compartment.^1^, ^2^ The same has been observed in the cells from affected individual A‐II‐2 from this study. Additionally, the trafficking defect upon BFA treatment is a specific feature for ATPase related CL condition, which is absent in other CL conditions (Table 1).^2^ This implies a functional defect of this compartment, potentially leading to secretion defects of extracellular matrix components and other factors. Interestingly, Ron Wevers and Eva Morava recently discovered a pathogenic variant in *PI4K2A*, encoding phosphatidylinositol 4‐kinase type 2‐alpha as the cause of a CL condition with strong neurological features, movement disorders and a marked myopathy.^31^ Golgi‐derived phosphoinositol‐4‐phosphate containing (PI(4)P) vesicles were recently described to be involved in mitochondrial fission processes.^32^ In light of this work, one might speculate that trans‐Golgi network dysfunction and intracellular trafficking problems due to loss of v‐ATPase components might secondarily affect mitochondrial function by a reduced availability of PI(4)P positive vesicles.

The identified compound heterozygous pathogenic variants in *ATP6V1A* have not been described previously and are absent from gnomAD and TGP. Additionally, they were predicted to be disease causing by different in silico prediction tools and the affected gene is in agreement with the phenotype leading to a classification as a pathogenic variant according to the ACMG criteria. The mutations p.(Met95Lys) and p.(Leu106Ser) affect highly conserved residues within the ATP6V1A polypeptide. The alteration p.(Met95Lys) resides within a loop whereas the substitution p.(Leu106Ser) affects the beginning of an alpha‐helix. Both substitutions change the properties on the respective position from a non‐polar amino acid to a polar one, very likely interfering with structural properties of the monomer. All variants in *ATP6V1A* causative for ARCL2D known so far are missense variants leading to an instable V_1_ sector.^1^ Therefore, it is likely that the herein identified missense substitutions might also cause an altered structure and thereby lead to a generalized assembly problem of this sub‐complex. The identified nonsense pathogenic variant is the first alteration of this kind detected in an individual with ARCL2D. The allele carrying the nonsense alteration completely underwent degradation as expected for this type of variant. In line with these data, also ATP6V1A protein expression was strongly reduced. The phenotype observed in *ATP6V1A*‐related CL affected individuals belongs to the most severe end of CL phenotypes associated with pathogenic variants in ATPase components. Therefore, and in line with the increased rate of early postnatal lethality, one might speculate that biallelic nonsense pathogenic variants in *ATP6V1A* are not compatible with life in humans.

Another feature affected individuals with CL and pathogenic variants in components of the v‐ATPase have is a congenital defect of glycosylation. In *ATP6V0A2* and *ATP6V1E1* related CL, this is a constant feature in the individuals tested; however, in *ATP6V1A* this seems to be variable (Table 1).^1^ In two affected individuals described by van Damme et al, this was once detectable while it was absent in another test. In proband A‐II‐1 from this study, transferrin isoelectric focusing did not show any glycosylation abnormalities. *ATP6V1A* pathogenic variants reduced the abundance of ICAM‐1 at the surface of fibroblasts, which is an indication for a glycosylation defect. We decided to analyze N‐glycosylation within the Golgi apparatus using a pulse chase experiment with a precursor for sialic acid. We found a significant reduction of sialylation within this compartment, comparable to what was seen in *ATP6V0A2* deficient fibroblast.^29^

In conclusion, we herein report on three additional affected individuals due to pathogenic variants in *ATP6V1A* as the cause for a severe cutis laxa phenotype. One of these variants is the first nonsense allele described in this gene. This study expands our knowledge about this extremely rare condition and the postnatal cardiac decompensation leading to death of one sibling stresses the fact that these patients may need neonatal intensive care.

## CONFLICTS OF INTEREST

Guido Vogt, Naji El Choubassi, Ágnes Herczegfalvi, Heike Kölbel, Anja Lekaj, Manuel Holtgrewe, Sabine Krause, Rita Horvath, Markus Schuelke, Christoph Hübner, Stefan Mundlos, Ulrike Schara, Andreas Roos, Hanns Lochmüller, Veronika Karcagi, Uwe Kornak, and Björn Fischer‐Zirnsak declare that they have no conflict of interest.

## AUTHOR CONTRIBUTIONS

Guido Vogt and Naji El Choubassi equally participated in acquisition of data, analysis and interpretation of the data and the drafting of the manuscript. Ágnes Herczegfalvi, Heike Kölbel, Anja Lekaj, Manuel Holtgrewe, Sabine Krause, Rita Horvath, Stefan Mundlos, Markus Schuelke, Christoph Hübner, Ulrike Schara, Andreas Roos, Hanns Lochmüller and Veronika Karcagi participated in acquisition of data, analysis and interpretation of the data. Uwe Kornak and Björn Fischer‐Zirnsak participated in the study concept and design, acquisition of data, analysis and interpretation of the data, the drafting of the manuscript and for important intellectual content. Björn Fischer‐Zirnsak supervised the study. All authors critical revised the manuscript.

## ETHICS STATEMENT

The Charité ‐ Universitätsmedizin Berlin ethics committee approved the study “Genetic Causes and Pathomechanisms of Cutis Laxa” (EA2/145/07).

## INFORMED CONSENT STATEMENT

All procedures followed were in accordance with the ethical standards of the responsible committee on human experimentation (institutional and national) and with the Helsinki Declaration of 1975, as revised in 2000 (5). Informed consent was obtained from all patients for being included in the study. Additional informed consent was obtained from all patients for which identifying information is included in this article.

## ANIMAL RIGHTS

This article does not contain any studies with human or animal subjects performed by the any of the authors.

## DATA AVAILABILITY

Anonymized data will be shared by reasonable request from any qualified investigator for purposes of replicating procedures and results.

## Supporting information


**Supplementary Figure 1** Histological and immunofluorescence findings from individual BII‐1: The periodic acid‐Schiff (PAS) reaction displayed deposits in a proportion of muscle fibers indicative for accumulation of polysaccharide‐structures (white arrows). Oil red staining identified muscle cells with sarcoplasmic lipid accumulation (white arrows). NADH‐reaction unraveled muscle fibers with reduced focal reactivity (white arrow). Immunofluorescence study of actin shows increased reactivity of in a minority of muscle cells (white arrow). Prominent build‐up of deposits of myotilin, filamin C and desmin in a proportion of muscle fibers are respectively highlighted (white arrows). Immunofluorescence studies of caveolin‐3 revealed an immunoreactivity of the rim of vacuoles (white arrows). Scale bar: 20 μm

**Appendix** S1. Tables
