# Supplementary material for: Expanding the clinical and molecular spectrum of ATP6V1A related metabolic cutis laxa
Source: J Inherit Metab Dis. 2021 Feb 4;44(4):972–86. doi: 10.1002/jimd.12341 (PMC8638669; doi:10.1002/jimd.12341)
Supplement: Supplementary file 1 — Supplementary Figure 1 Histological and immunofluorescence findings from individual BII‐1: The periodic acid‐Schiff (PAS) reaction displayed deposits in a proportion of muscle fibers indicative for accumulation of polysaccharide‐structures (white arrows). Oil red staining identified muscle cells with sarcoplasmic lipid accumulation (white arrows). NADH‐reaction unraveled muscle fibers with reduced focal reactivity (white arrow). Immunofluorescence study of actin shows increased reactivity of in a minority of muscle cells (white arrow). Prominent build‐up of deposits of myotilin, filamin C and desmin in a proportion of muscle fibers are respectively highlighted (white arrows). Immunofluorescence studies of caveolin‐3 revealed an immunoreactivity of the rim of vacuoles (white arrows). Scale bar: 20 μm [file JIMD-44-972-s002.pdf]

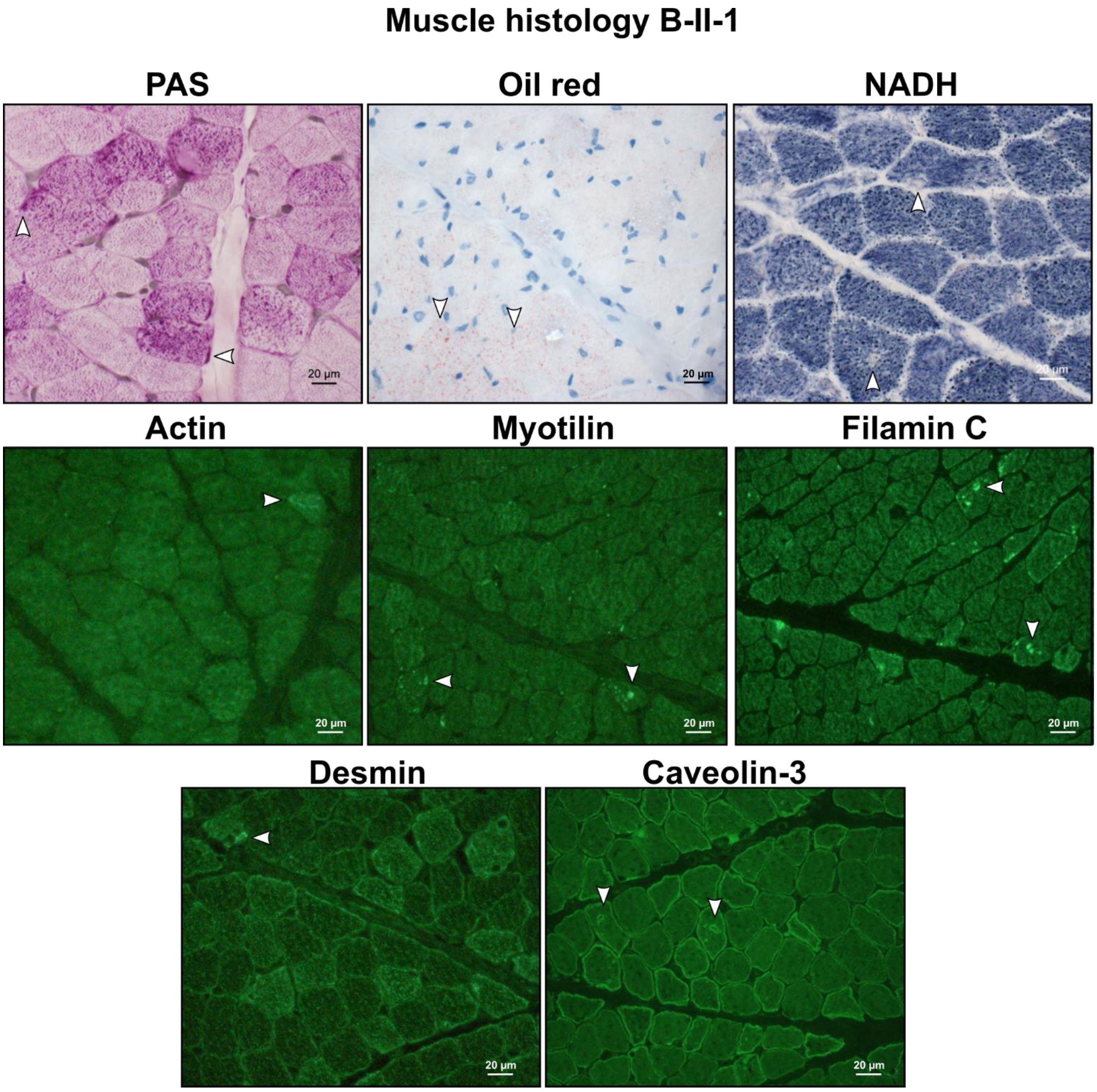

Supplementary Figure 1: Histological and immunofluorescence findings from individual B-II-1: The periodic acid–Schiff (PAS) reaction displayed deposits in a proportion of muscle fibres indicative for accumulation of polysaccharide-structures (white arrows). Oil red staining identified muscle cells with sarcoplasmic lipid accumulation (white arrows). NADH-reaction unraveled muscle fibres with reduced focal reactivity (white arrow). Immunofluorescence study of actin shows increased reactivity of in a minority of muscle cells (white arrow). Prominent build-up of deposits of myotilin, filamin C and desmin in a proportion of muscle fibres are respectively highlighted (white arrows). Immunofluorescence studies of caveolin-3 revealed an immunoreactivity of the rim of vacuoles (white arrows). Scale bar: 20 µm
