# Supplementary material for: Expanding the clinical and molecular spectrum of ATP6V1A related metabolic cutis laxa
Source: J Inherit Metab Dis. 2021 Feb 4;44(4):972–86. doi: 10.1002/jimd.12341 (PMC8638669; doi:10.1002/jimd.12341)
Supplement: Supplementary file 2 — Appendix S1. Tables [file JIMD-44-972-s001.docx]

| ***ATP6V1A* genomic DNA primers Exon 4 & Exon 13** | |
| --- | --- |
| Exon 4 LEFT PRIMER | TGCTACTCAGTTGTTAGGACGT |
| Exon 4 RIGHT PRIMER | AGTTCCTCTGTTTCGTGGGG |
| Exon 13 LEFT PRIMER | TTGCCACCATCAATCTTACA |
| Exon 13 RIGHT PRIMER | CCTTCACCAAAACACAACAGA |

Primer sequences used for validation and co-segregation analyses.

| **cDNA sequencing of *ATP6V1A* Exon 4 – Exon 13** | |
| --- | --- |
| hseqATP6V1A Exon 4 F | CGCACTGGTAAACCCCTCTC |
| hseqATP6V1A Exon 4 R | TTGCAAGGTGTAAAGTCCCA |
| hseqATP6V1A Exon 13 F | GTTCGTTCCTCTGAGGACGA |
| hseqATP6V1A Exon 13 R | ACAGCATCCCTACTGTCTTGT |

Primer sequences used for sequencing the variants c.317T>C and c.1513_1514del in *ATP6V1A on cDNA*

| **qPCR primers *ATP6V1A* Exon 1-2** | |
| --- | --- |
| qhATP6V1A Ex1-2 F: | TGGTTATGTGCATGGGGTCT |
| qhATP6V1A Ex 1-2 R: | AGTAGCCATGTCACCCTCCA |

Primer sequences used for quantitative RT-PCR to determine *ATP6V1A expression*
